# Why, when and how to update a meta-ethnography qualitative synthesis

**DOI:** 10.1186/s13643-016-0218-4

**Published:** 2016-03-15

**Authors:** Emma F. France, Mary Wells, Heidi Lang, Brian Williams

**Affiliations:** Nursing, Midwifery and Allied Health Professions Research Unit, School of Health Sciences, University of Stirling, Stirling, FK9 4NF UK

**Keywords:** Cancer, Qualitative analysis, Meta-ethnography, Meta-synthesis, Systematic reviews, Qualitative reviews

## Abstract

**Background:**

Meta-ethnography is a unique, systematic, qualitative synthesis approach widely used to provide robust evidence on patient and clinician beliefs and experiences and understandings of complex social phenomena. It can make important theoretical and conceptual contributions to health care policy and practice.

**Results:**

Since beliefs, experiences, health care contexts and social phenomena change over time, the continued relevance of the findings from meta-ethnographies cannot be assumed. However, there is little guidance on whether, when and how meta-ethnographies should be updated; Cochrane guidance on updating reviews of intervention effectiveness is unlikely to be fully appropriate. This is the first in-depth discussion on updating a meta-ethnography; it explores why, when and how to update a meta-ethnography. Three main methods of updating the analysis and synthesis are examined. Advantages and disadvantages of each method are outlined, relating to the context, purpose, process and output of the update and the nature of the new data available. Recommendations are made for the appropriate use of each method, and a worked example of updating a meta-ethnography is provided.

**Conclusions:**

This article makes a unique contribution to this evolving area of meta-ethnography methodology.

**Electronic supplementary material:**

The online version of this article (doi:10.1186/s13643-016-0218-4) contains supplementary material, which is available to authorized users.

## Background

Qualitative syntheses of multiple individual qualitative studies provide robust evidence to inform health care policy and practice [1, 2]. Meta-ethnography [3], an inductive, interpretive approach upon which most interpretive qualitative synthesis methods are based [4], is the most commonly used qualitative synthesis approach in health-related research [5]. It is particularly suited to developing conceptual models and theories. Noblit and Hare [3], ethnographers in education research, developed meta-ethnography in the 1980s to deal with synthesising contradictory concepts from interpretive study accounts with unique contexts.

There are numerous qualitative synthesis approaches [6, 7] which differ in their purposes, philosophical traditions and whether they primarily aggregate or re-interpret (“re-configure”) study findings [8]. Unlike other qualitative synthesis approaches, in a meta-ethnography, the reviewer re-interprets the conceptual data, i.e. themes, concepts or metaphors, created by the authors of primary study accounts using a unique synthesis method (described below) in order to transcend the findings of individual study accounts [3].

Noblit and Hare [3] described seven phases of a meta-ethnography:

1. “Getting started”—choosing the topic focus. The focus may evolve through reading of study accounts.
2. “Deciding what is relevant to the initial interest”—identifying and selecting study accounts to synthesise. This need not involve exhaustive systematic searches for studies [3].
3. “Reading the studies”—repeated reading of study accounts and detailed recording of the concepts, themes and metaphors.
4. “Determining how studies are related”—comparing study accounts by creating and juxtaposing a list of concepts from each study to judge whether the concepts are similar, contradictory, or about different aspects of the topic being researched. This indicates which type of synthesis is possible: “reciprocal”, “refutational” or a “line of argument synthesis”, respectively.
5. “Translating the studies into one another”—systematically comparing or “translating” themes, metaphors or concepts across and within primary study accounts. This is not literal translation but translation of meaning to generate explanation of a phenomenon. It is a process akin to constant comparison (systematically comparing all the data throughout the analysis process). Translation is based upon Turner’s theory of social explanation [9] which maintains that all social explanation is comparative and must be inductive. The translated concepts collectively are one level of synthesis. The translation process is key to meta-ethnography.
6. “Synthesising translations”—when phase 5 results in many translations, these can be compared to see if there are common types of translations or if some translations or concepts can encompass those from other study accounts to reach new interpretations.
7. “Expressing the synthesis”—conveying the findings of the synthesis in a form suitable for the particular audience.

Meta-ethnographies can refute or revise understanding of a phenomenon [10]; generate testable models, theories and hypotheses [11]; provide a historical overview of concepts or theories [10]; increase the relevance of findings from single qualitative studies for broader contexts [12]; identify directions for future research; reveal when no new conceptual development in a field has occurred [13]; inform and enhance the design of complex interventions; and enhance interpretation of systematic reviews of intervention effectiveness [14]. Consequently, qualitative syntheses are increasingly used to provide evidence to underpin health care policy and practice, for example, by Cochrane [15], the World Health Organization (http://optimizeMNH.org) [15] and the National Institute for Health and Care Excellence (NICE) [16].

Evidence syntheses of any kind can become out-of-date. However, there is very little guidance relating to whether, when and how to update a qualitative synthesis [17]. In this paper, our aim is to contribute to the limited published literature on the process of updating a meta-ethnography which is particularly challenging because of its unique and complex synthesis process. First, we explore why and when a meta-ethnography (or other qualitative synthesis) should be updated; then, we identify how to do it, critiquing different methods of updating a meta-ethnography; and finally, we give a worked example of how we updated a meta-ethnography on experiences of head and neck cancer (HNC).

## Results

### Why and when to update a meta-ethnography

Qualitative syntheses can become out-of-date because beliefs, experiences and social phenomena change over time, but there is no published guidance on why and when to update any type of qualitative synthesis [17]. Cochrane specify that systematic reviews of intervention effectiveness should be updated every 2 years [18], unless their quality and current relevance can be assured [19]. Cochrane guidance suggests reviewers consider how time-dependent the review findings are, based on, for example, the availability of new evidence, new health care treatments and/or advances in review methodology [18]. However, it is unclear to what extent reviewers can apply this guidance to qualitative syntheses; furthermore, evidence on the optimum timeframe for updating systematic reviews of interventions is sparse [20].

There is a range of reasons why an update of a qualitative synthesis might be necessary. These include the purpose, quality and time-dependency of the original meta-ethnography and the volume and content of new, relevant qualitative studies. We now explore each of these reasons. If the aim of the original meta-ethnography was tied to a specific time period in the past (for example, the effect of a previous policy), then the meta-ethnography findings may still be relevant to that time. However, the same findings might not be useful for current policy and practice if important new issues are omitted or they focus on outdated practices. As with quantitative reviews, there is no fixed time interval after which a meta-ethnography becomes out-of-date. Important considerations include the rate at which new qualitative evidence is being published and the content of the published studies.

A high volume of new published studies could warrant an update. Ideally, new studies should contribute novel concepts to the original meta-ethnography, add depth to concepts already identified or show that concepts already identified also apply to new health care settings, interventions, patient populations or treatments. Therefore, a synthesis that reached “conceptual saturation” (when no further concepts are generated by including in the analysis data from more articles with similar populations) might not become out-of-date rapidly; inclusion of further studies is unlikely to add insights. However, if there were few studies or concepts in the original synthesis or if new studies contain novel data on people’s experiences, then an update is desirable.

If the original meta-ethnography is of low quality then a rigorous, well-conducted update could increase the trustworthiness and hence the utility of its findings. Quality judgements should be based on the conduct and/or reporting of the meta-ethnography or the existence of new methodological advances in meta-ethnography or qualitative reviewing. In terms of timing, updating a poor quality meta-ethnography could be done as soon as a quality issue is identified, regardless of the existence of new relevant publications.

**Fig. 1:**
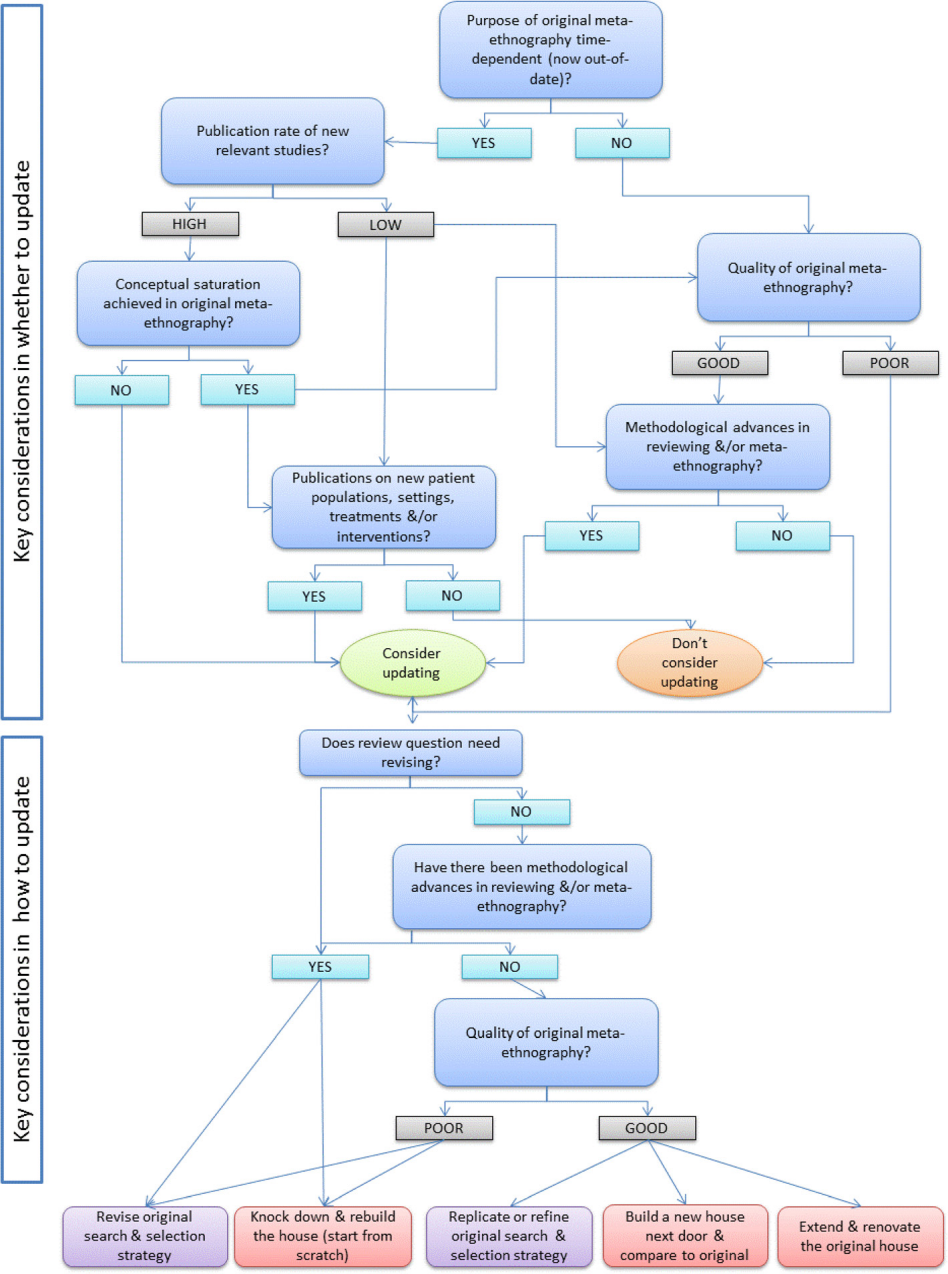
Flow chart of the decisions whether and how to update a meta-ethnography

Out-of-date qualitative syntheses may have negative consequences: health promotion interventions based on “old” syntheses of data on experiences may no longer be relevant and effective (for example, they may inadvertently encourage undesirable health behaviours) and could even stigmatise a condition (such as HIV/AIDS awareness advertising campaigns from the 1980s which emphasised its incurability and deadliness). The design of services may reflect outmoded attitudes and beliefs and thus offend people leading to reduced engagement with services, for example, cancer services in the past did not always directly disclose a cancer diagnosis to patients which would be viewed as unacceptable practice in the UK today. We summarise the possible consequences of outdated syntheses in Table 1 and provide guiding questions to help reviewers determine if and when an update is warranted (see also Fig. 1).

**Table 1:** Questions to guide whether, when and how to update a meta-ethnography

Guiding questions	Issues to consider	This is a consideration in deciding		
		Whether to update	When to update	How to update
Q1. What was the aim of the original meta-ethnography (and is it time-sensitive)?	If data are out-of-date, then an update might be needed. If the aim was tied to a specific time period in the past, the original findings may not be relevant to current practice. The aim may have implications for how to update, given the original search strategy.	✓		✓
Q2. What is the aim of the updated meta-ethnography?	The original and updated search strategies must be compatible with the update aim.			✓
Q3. What is the publication rate of relevant new studies?	A high volume of publications could warrant an update and influence how the update is done (see also Q6).	✓	✓	✓
Q4. What is the quality of the (conduct and reporting) of the original meta-ethnography?	If it is low quality: • an update could increase the trustworthiness and hence utility of the findings • the value of adding to or comparing with the original findings is questionable • in terms of timing it could be re-done immediately.	✓	✓	✓
Q5. Have there been methodological advances in qualitative reviewing and/or the meta-ethnography approach?	If yes, an update could increase the trustworthiness and hence utility of the findings. In terms of timing, it could be re-done immediately (see also Q6).	✓	✓	✓
Q6. Was conceptual saturation achieved in the original meta-ethnography?	If yes, inclusion of further relevant studies is unlikely to add insights unless they contain new data on experiences (see Q7).	✓		
Q7. Are there (publications on) new patient populations, health care contexts, treatments and/or interventions since the original meta-ethnography?	If yes, an update could be useful because people’s experiences might differ from those already reported. Could require a revised review question and literature search strategy.	✓	✓	✓
Q8. Does the review question need to be revised?	If answer to Q7 is “yes”, then it may be necessary to revise the review question, literature search strategy and inclusion criteria, e.g. to include a new patient population.	✓		✓
Q9. What are the potential consequences of not updating the original meta-ethnography?	If findings are out-of-date but being applied in current policy/practice, the findings may not be useful or may even be harmful.	✓

### How to update a meta-ethnography—literature searches

Once reviewers decide to update a meta-ethnography, they should consider two main methodological processes: (1) how to revise the literature searches and study selection and (2) how to perform the analysis and synthesis. In Table 1, we outline factors that impact on how an update should be conducted, and Fig. 1 illustrates how to choose update methods. There are two main options when updating searches and selection: replicate the earlier strategy or revise it in line with the aim of the update or a modified review question or study inclusion criteria—comprehensive searches may be unnecessary. New patient populations, health care settings, treatments and/or interventions since the original meta-ethnography could require a revised review question and literature search strategy to enable identification and inclusion of new relevant literature. Searches can be either comprehensive or selective, and further purposive selection of publications may be appropriate, depending on the aim of the synthesis and update.

Daker-White et al. [21] updated their published meta-ethnography of the experience of living with rheumatoid arthritis by doing a separate new meta-ethnography. Their aim was to examine conceptual development over time. However, they scaled down their original comprehensive search strategy because of resource limitations. The original strategy had involved comprehensive searches of seven databases, hand searching 11 journals, back searching reference lists of included papers and inclusion of grey literature. For the update, they conducted a simple search of only one database and did back searching. Consequently, their original and updated meta-ethnographies did not contain comparable bodies of literature; it is possible they may have missed contradictory studies which could weaken their conclusion that newer studies confirmed earlier ones [21].

We do not recommend scaling down literature searches for an update without a strong rationale. Doing a less, rather than more, comprehensive search might be justifiable in certain circumstances. For instance, if (after comprehensive searches) reviewers are confident that they had reached conceptual saturation in their original meta-ethnography, their update could strategically search for and purposively sample only studies likely to provide new data on experiences, for instance, studies involving new patient populations. Increasingly qualitative reviewers regard exhaustive searching as unnecessary for qualitative syntheses whose purpose is to develop knowledge and theory [22]. In brief, the search strategy should suit the aim of both the original meta-ethnography and the update. Amending the original literature searches has implications for deciding how to update the analysis and synthesis; we discuss this below.

### How to update a meta-ethnography—analysis and synthesis

We can find no published guidance on how to update the analysis and synthesis for a meta-ethnography. Nonetheless, based on our experiences of and reflections on updating our own syntheses and three published updates [23–25], we identified three possible methods:

1. Add to and revise the existing meta-ethnography to incorporate the new articles. To use an analogy related to house building, this would be like extending and renovating the original house.
2. Do a new, standalone synthesis of the new articles, then compare the findings to the original meta-ethnography. To continue the analogy, this would be like building a new house next door to the original and comparing the two houses.
3. Start the analysis and synthesis again from “scratch” (from the beginning) by incorporating the older articles with the newer ones to create a single overarching synthesis. This would be equivalent to knocking down the house and rebuilding it.

#### Extend and renovate the original house

We identified two published journal articles [24, 25] - a meta-ethnography of stakeholder experiences and perceptions of tuberculosis and treatment [25] and one on experiences and perceptions of breastfeeding support [24]—which described their approach to updating the analysis and synthesis. The descriptions were extremely brief: “data were then juxtaposed alongside the original synthesis to compare ‘fit’ and examined for new emerging themes” [25] (p. 231); and “articles were reviewed by one author […] and the themes compared to those in the original report. New themes which emerged were noted.” [24] (p. 417). Reviewers in both articles examined if the newly identified studies added new concepts or contributed to existing ones to create a new single, overarching synthesis. We also identified a NICE clinical guideline [23], which described in more detail updating an existing meta-ethnography through revising it. However, rather than using meta-ethnographic methods they did this by conducting an aggregative narrative synthesis, which we considered potentially incompatible with the interpretive meta-ethnography approach since aggregation would limit the reviewers’ ability to enhance or refine the original theory or interpretations.

There are several possible advantages of updating a meta-ethnography by extending and renovating—the output is in the form of one coherent model or set of findings, rather than two, which can increase its usefulness for the end user; there is no arbitrary dividing date between the literature in the original and update; and it is an efficient use of the resources expended on conducting the original synthesis to build on it. Disadvantages could include: update findings might be constrained or influenced by the original findings, especially if done by the original reviewers; it is challenging for a new team of reviewers if they do not have full access to the prior analysis; and there are a lack of established methods for updating in this manner. It is not ideally suited to comparing two bodies of literature over time, is of questionable value if the original review is low quality, and possibly inappropriate if the review question or literature searches have been revised. We summarise the advantages and disadvantages of this and other methods of updating in Table 2.

**Table 2:** Summary of characteristics of different methods of updating the analysis and synthesis in a meta-ethnography

	Method of updating the analysis and synthesis in a meta-ethnography		
Characteristics	Extend and renovate the house (add to and revise original)	Build a new house next door (do a new standalone meta-ethnography and compare to original)	Knock the house down and rebuild it (start again from scratch)
Possible advantages			
One coherent model, set of findings, conclusions (increases utility of output for end users)	✓		✓
Can lead to new conceptual insights	✓	✓	✓
Allows innovation in analysis/synthesis process in update	✓	✓	✓
No arbitrary dividing date between literature in original and updated meta-ethnographies	✓		✓
Efficient use of resource expended on original meta-ethnography	✓	✓	
Facilitates comparisons between two sets of literature from different time periods		✓	
More easily done by a new team of reviewers		✓	✓
Can implement methodological advances in meta-ethnography/qualitative reviewing			✓
Can improve quality and utility of poor quality original meta-ethnography			✓
Suitable if you have revised review question or study selection criteria			✓
Possible disadvantages			
Challenging for a new team of reviewers	✓		
Update findings might be influenced by original findings, especially if done by original reviewers	✓	✓	✓
Can minimise influence of findings from original meta-ethnography, especially if done by new reviewers			✓
Lack of established methods for updating original analysis/synthesis	✓		
More likely to have large number of articles to synthesise (>40 is challenging)			✓

#### Build a new house next door to the original and compare the two houses

Since we updated our meta-ethnography on HNC, two more updated meta-ethnographies [17, 21] have been published in which reviewers conducted a separate synthesis of new studies and compared this to their original meta-ethnography. McCann, Campbell and Entwistle [17], when updating their meta-ethnography on the reasons why patients participate in clinical trials, reported that this method enabled them to innovate in their analysis process by grouping studies according to the type of health issue prior to synthesis. The authors stated that this process led to new insights and a more complex model compared to their original meta-ethnography. They reported the comparison of their two meta-ethnographies by briefly summarising the key findings and conceptual model from their original, presenting the methods and results of their update only and indicating in one sentence how the two syntheses differed:

Our two meta-ethnographic syntheses were broadly compatible, but the second more clearly indicated how participants’ health-related situations can mediate the significance for people’s decisions about participation of key features of recruitment processes (including relationships with trial staff), of the interventions and processes involved in particular trials, and of participants’ general inclinations to support research. [18] (p. 239).

They chose not to add to their original meta-ethnography because they wanted to avoid forcing more recent data to fit their original findings; they purposely did not refer to the findings of their original until the update had been completed. In retrospect, they concluded their concerns had been unfounded since their two meta-ethnographies had broadly similar findings. They reflected that their update approach had aided identification of the insights added by the newer studies.

Daker-White et al. [21] chose the same update approach to address their aim of illustrating conceptual development through time. In their original and updated meta-ethnographies of experiences of rheumatoid arthritis, they compared studies published in two time periods; however, these periods were arbitrarily separated by the date on which they had conducted the original literature searches. This typical approach to updating literature searches perhaps limits the usefulness of the “build a new house next door” method of updating unless, by chance, the newer studies all have a different focus on the same topic than the older studies. Daker-White et al. [21] presented the methods and findings of their two syntheses separately and then compared the key concepts from each. They also compared the focuses, sorts of coping strategies identified, study designs, academic disciplines of the authors and the conceptual depth in the two bodies of literature. They concluded that in the update: “papers were more concerned about perceptions of ‘control’ and incorporated a ‘career’ model of the experience of RA [rheumatoid arthritis] where people adapted to new circumstances brought by the disease.” [21] (p. 8).

A possible disadvantage of doing a new standalone meta-ethnography is that the findings and models from the original and the update might not be fully integrated, so making it harder for end users to use the output. Also, if the original meta-ethnography was of low quality (or used incompatible literature search and selection methods), then the value of comparing it with the update might be questionable.

#### Knock down the house and rebuild it

An alternative method of updating a meta-ethnography, not reported in published meta-ethnographies, is to start again from scratch. This involves reviewers “knocking down and rebuilding the house” by integrating the old and new articles into a single new meta-ethnography, disregarding the earlier findings. This may or may not involve re-designing and re-running the original literature search and selection strategy. Starting again could be necessary in certain circumstances, also described in Table 2, for instance, if the original meta-ethnography was poor quality in conduct or reporting, if the reviewers have revised the review question or study selection criteria or if they need to implement methodological advances in meta-ethnography or qualitative reviewing.

Starting from scratch may be desirable to avoid the updated findings being influenced or constrained by the original findings; to avoid having an arbitrary dividing date between the bodies of literature in the original and updated meta-ethnographies; to allow innovation in the analysis and synthesis process in the update; and/or to produce one coherent model or set of findings to increase the utility of the output for end users; otherwise, it may be more efficient to build upon (extend) the existing meta-ethnography. However, if the overall number of published studies to be synthesised is high then starting again from scratch might be unfeasible, unless purposive sampling is used. Some expert reviewers believe that around 40 articles is the maximum number that can be synthesised effectively using the meta-ethnography approach [13], but over 70 articles have been synthesised successfully [26]. Next, we report how we approached an update of our meta-ethnography [27] to examine patients’ experiences of HNC, focusing on the analysis and synthesis processes.

### A worked example of updating a meta-ethnography

In 2007, we conducted a meta-ethnography on patients’ experiences of HNC which was, after only a few years, potentially out-of-date: we were aware that demographic changes in the HNC population and the increasing use of complex multi-modality treatments might provide important new insights; it was likely that these new populations and treatments would be covered by the relatively large number of new relevant publications. Therefore, we decided to update our original meta-ethnography, which we did in 2011 and 2012. We intended to replicate our original exhaustive systematic search and selection strategy to help ensure identification of all relevant articles. In our original meta-ethnography, we had included 15 articles published from 1993 to September 2007. In our update, we identified a further 14 relevant articles published from September 2007 to September 2011.

There were no apparent reasons to suspect that our original meta-ethnography was of poor quality—it had been conducted for a doctoral thesis using a rigorous approach involving three senior qualitative researchers and one more junior researcher. Also we were not aware of recent relevant methodological advances and the date dividing our literature searches was arbitrary; therefore, we chose to “extend and renovate” our original meta-ethnography. We have reported the methods in detail for the updated meta-ethnography in Lang et al. [27]; here, we focus on the analysis and synthesis processes.

It was unclear exactly how previous reviewers [24, 25], who had used a similar update approach, had done this and so we had no methodological guidance to assist us. In our original meta-ethnography, we had produced six final synthesised concepts, described in Table 3. To make the most efficient and effective use of our previous analytic work, we decided to build on, rather than disregard, our original analysis by “extending and renovating the house”. We had to adapt Noblit and Hare’s [3] analysis and synthesis processes (phases 4 to 6), which were designed for conducting a standalone meta-ethnography, to conduct the complex process of updating a fully formulated synthesis. We describe below and in Table 4 and Additional file 1: Table S1 how we revised our analysis and synthesis, including how we incorporated contradictory concepts.

**Table 3:** Six synthesised concepts from original and updated meta-ethnography

Translated concepts from phase 5 of the original	Translated concepts from phase 5 of the update	Final synthesised concepts from phase 6 of original & update (encompassing all translated concepts)	Synthesised concept description in original meta-ethnography	Synthesised concept description in updated meta-ethnography
• Living and waiting		Uncertainty and waiting	Being in limbo—the uncertainty of living with the disease and of the future.	Being in limbo—the uncertainty of living with the disease and of the future.
• Disruption to life and living • The experience of symptoms	• The experience of diagnosis	Disruption to daily life	The disruption of treatment to the patient's physical functioning, emotions and social life.	Patients experience disruption in all aspects of life because of the effects of cancer and its treatment, beginning with the shock of diagnosis. After diagnosis, life is disrupted physically, emotionally and socially.
• Enduring or moving on • The diminished self		The diminished self	The temporary or longer-lasting functional, social and existential losses patients experience and the impact of these.	Patients experience temporary or longer-lasting functional, social and existential losses, which can alter their life expectations. The stigma of changed appearance and speech, damaging experiences with health care professionals (HCPs), and perceived rejection by their next of kin contribute to losses.
• Information • Fears and expectations • The significance of symptoms	• Explaining HNC to family and children • Seeking cause of HNC • No choice – treatment or death	Making sense of the experience	Patients' continual efforts to make sense of cancer and what is happening to them and how they develop expectations about a likely outcome.	Patients' continual efforts to make sense of their cancer and what is happening to them and to help their family - including their children - to make sense of their illness.
• Connection with HCPs • Communicating the hidden experience	• Connection with family and social network • Connection with peers with head and neck cancer (HNC)	Sharing the burden	The importance of a supportive relationship with HCPs whose role is crucial in instilling hope, maintaining self-worth and counteracting patients' vulnerability.	Developing supportive connections with family, friends, their wider social network, HCPs and other people with HNC helps patients to cope emotionally and practically with their illness.
• Finding ways to deal with an uncertain future	• Enhanced future • Coping with dying • Self-management	Finding a path	Reflects the nature of life beyond cancer. Patients perceive their future as either diminished or changed.	Reflects how patients characterise life beyond HNC. Some limit their focus to the present, living in the here and now, particularly when cancer is terminal. Others perceive their future as either diminished, changed or enhanced. Establishing successful coping and self-management strategies is associated with perceiving a changed or enhanced future.

Adapted from Lang et al.^20^

**Table 4:** Summary of the phases of meta-ethnography conduct and updating

Noblit and Hare’s [3] 7 phases	How we conducted each phase in our original HNC meta-ethnography	How we conducted each phase in our updated HNC meta-ethnography
1. “Getting started” (the topic focus).	To examine patients’ experiences of HNC to provide a context for future research.	To examine patients’ experiences of HNC to provide a context for future research.
2. Deciding what is relevant to the initial interest.	Exhaustive systematic search strategy; inclusion of qualitative studies of the experience of HNC up to September 2007. Included 15 articles.	Replicated earlier search strategy and inclusion criteria from September 2007 to September 2011. Identified a further 14 relevant articles.
3. Reading the studies.	We identified, recorded and described on index cards all the primary study authors’ concepts and main conclusions in the 15 articles.	We identified, recorded and described on index cards the primary study authors’ concepts and main conclusions in the 14 articles.
4. Determining how studies are related.	We directly compared the primary study authors’ concepts and found them to be reciprocal (about roughly similar things).	We juxtaposed the primary study authors’ concepts from each new article with our 11 translated concepts from phase 5 of the original to compare meanings. Most concepts from the new articles were reciprocal, but some were contradictory.
5. Translating the studies into one another.	We systematically compared the meanings of all the primary study authors’ concepts across the articles and grouped the concepts according to shared meaning through reciprocal translation to produce 11 translated concepts.	We continued the original translation process by systematically comparing the meanings of the primary study authors’ concepts from each new article with our 11 translated concepts from the original. Most concepts confirmed or enhanced the original translated concepts. We developed 9 additional translated concepts. We re-examined the articles in the original meta-ethnography to check if they did in fact support the new issues and concepts.
6. Synthesising translations.	We compared and contrasted our 11 translated concepts and found that some could encompass or were similar to others resulting in a final six synthesised concepts (“synthesised translations”): uncertainty and waiting, disruption to daily life, the diminished self, making sense of the experience, sharing the burden, and finding a path.	We juxtaposed the 11 original and 9 new translated concepts with our six synthesised concepts from phase 6 of the original meta-ethnography to systematically compare meanings. We refined our synthesised concepts to reflect the new and contradictory concepts.
7. Expressing the synthesis.	In written form in an unpublished doctoral thesis.	In written and diagrammatic form in a published journal article.

#### Conducting the analysis and synthesis for the original meta-ethnography

In phase 3 (reading the studies) of our original meta-ethnography, we identified, recorded and described on index cards all the primary study authors’ concepts and main conclusions in the 15 articles. In phase 4 (determining how the studies are related), we directly compared the study authors’ concepts and found them to be reciprocal (about roughly similar things). In phase 5 (translating the studies into one another), we systematically compared the meanings of all the primary study authors’ concepts across the papers and grouped the concepts according to shared meaning through reciprocal translation—a process akin to constant comparison—to produce 11 translated concepts. Then, in phase 6 (synthesising translations) of the original, we further compared and contrasted these 11 translated concepts and found that some could encompass or were similar to others resulting in a final six synthesised concepts (“synthesised translations”).

#### Updating the analysis and synthesis

In the update, we repeated the same process for phase 3 as in our original meta-ethnography but our methods for phases 4 to 6 differed somewhat. In phases 4 and 5, which overlapped, we juxtaposed the primary study authors’ concepts from each new article with our 11 translated concepts from phase 5 of the original to systematically compare meanings. We asked if the primary study authors’ concepts from the new studies represented the same meaning, added new meaning or refuted the meaning of our original translated concepts. Most concepts from the new articles were confirmatory (reciprocal), but some were contradictory (refutational). Through continuing the reciprocal translation process, continuously comparing and grouping newly identified concepts according to shared meaning, we developed nine additional translated concepts not identified in the original, shown in Table 3. We also identified new aspects and issues relevant to our original 11 translated concepts and revised these, for example, “the diminished self” encompassed and was enhanced by new concepts concerning social stigma, perceived rejection by family members due to disfigurement and disability, and damaging experiences with health care professionals. We re-examined all the articles in the original meta-ethnography to check if they did in fact contain any previously unidentified data supporting the new and revised translated concepts.

In phase 6 of the update, we explored whether the revised original and nine additional translated concepts represented the same meaning, added new meaning or refuted the meaning of our original six synthesised concepts—we provide some examples of this process below. We concluded that the revised original and the nine new translated concepts in the new articles confirmed and supported the meaning of our six original synthesised concepts but added nuances or depth to five of them without changing their fundamental meaning, as seen in Table 3. Consequently, we then refined our original six synthesised concepts by adding further detail and concepts to them to reflect the new concepts from the more recent literature. Our updated synthesised concepts accommodated both confirmatory and contradictory concepts. In Additional file 1: Table S1, we describe the confirmatory, new and contradictory data or concepts from each new article.

#### Examples of analysis and synthesis in the update

An example of contradictory concepts in newer articles related to people’s perceptions of an enhanced self or future after HNC diagnosis and treatment (for example, because their family relationships had improved), which contrasted with concepts in the older articles about people’s sense of self being damaged. These new contradictory concepts formed the new translated concept an “enhanced future”. After reflection, we concluded that perceiving an enhanced self or future reflected a way of moving on with life, so we included this as a new facet of the synthesised concept “finding a path”, which was originally about how people dealt with life beyond cancer. Similarly, we decided that the translated concept of self-management (for example, establishing healthy eating habits) in newer articles belonged in “finding a path” because it described people adapting to a changed future. Table 3 shows all the translated concepts from the original and update and which final synthesised concepts encompassed them.

Another example of a refutational concept was the apparently positive interpretations of experiencing advanced stage HNC in the newer articles. For instance, three articles contained similar concepts about some people’s “positive” reactions to dying: they involved people coping by planning ahead for death, placing limits on their life or focusing on the present. These concepts formed a new translated concept “coping with dying” which added new dimensions, relating to people using coping strategies and living in the present, to the synthesised concept “finding a path,” which in the original meta-ethnography was about people seeing their future as diminished or changed.

## Discussion

We have presented a worked example of one possible method for updating a meta-ethnography. If we had used a different update method or approached the analysis and synthesis process differently (for example, if we had disregarded our 11 original translated concepts), we might have produced different findings because we might have noticed different conceptual groupings. Nonetheless, we believe that the final concepts resulting from a different analysis and synthesis process would have had equivalent meaning to ours, as McCann et al. [17] found in their build-a-new-house-next-door approach. Had we used McCann et al.’s approach in our update, then our final concepts might have differed given the greater focus on benefit finding and self-management in those studies. We feel this could have resulted in a synthesis that might have overstated the positives of living with HNC. However, future methodological research could formally compare and explore different ways of updating the analysis and synthesis process.

Many of the advantages and the pitfalls we have outlined apply to all three methods of updating a meta-ethnography but to differing degrees. For instance, when using any of the updating methods the new analysis and synthesis could be influenced by the findings of the original meta-ethnography, even if conducted by new reviewers. However, with the extending-and-renovating-the-house approach, there may be a greater danger of using a more deductive synthesis approach that could limit potential new insights, even if conducted by new reviewers. Other considerations apply mainly to one method, for example, extending and renovating the house (adding to and revising the original meta-ethnography) might make the most efficient use of previous analytic synthesis work and gives a fully integrated, single set of findings, whereas knocking down and rebuilding the house (starting again from scratch) is the most suitable method when there are concerns over the quality of the original.

There is no specific guidance for judging the quality of a meta-ethnography’s conduct or reporting, although a reporting guideline [28] and a method for assessing confidence in the findings of qualitative syntheses are being developed [29]. However, we believe that generally accepted quality indicators for any kind of qualitative study, such as its trustworthiness [30], apply also to a meta-ethnography. Transparent reporting is a pre-requisite to judging trustworthiness [31].

Doing a new overarching meta-ethnography (knocking down and rebuilding the house) could potentially change the findings of the original meta-ethnography more than if you added to it. However, it is probably unfeasible for reviewers to forget their original findings; therefore, completely re-doing a meta-ethnography might be easier for a new team of reviewers. All of the update examples cited in this article, apart from the NICE update, involved some of the same reviewers as the original. It is fairly common for new reviewers to update quantitative systematic reviews and this could become increasingly common for qualitative syntheses too, but it may be difficult for a new team to fully access the original team’s analysis to extend and renovate it.

Sensitivity analyses have been conducted for other qualitative synthesis approaches [32, 33] to evaluate the impact of inadequately reported primary studies on the synthesis findings. Future methodological work could explore the use of sensitivity analyses for meta-ethnographies as a means to evaluate the conceptual contribution of newer articles to the synthesis when using the extending-and-renovating-the-house approach.

## Conclusions

We have explored the reasons for why and when to update a qualitative synthesis and examined different methods of conducting an update of a meta-ethnography. We have developed some guiding principles for choosing a particular method and give a worked example of updating a meta-ethnography. We have developed questions to be used in combination with Tables 1 and 2 and Fig. 1 to guide those appraising updates to meta-ethnographies: is the reason for updating clear and justified? Is the aim of the update clear? Do the methods of updating the literature searches and selection and the analysis and synthesis fit the aim of the update? Does the search and selection strategy in the original meta-ethnography fit the aim of the update? There is very little published guidance on updating a meta-ethnography, and we believe this paper makes a unique contribution to this evolving area of meta-ethnography and qualitative synthesis methodology.

## Additional file

Additional file 1: Table S1.
**Fit between concepts in new papers and synthesised concepts from original meta-ethnography.**

## Abbreviations

HIV/AIDS: human immunodeficiency virus/acquired immune deficiency syndrome
HNC: head and neck cancer
NICE: National Institute for Health and Care Excellence
