# Supplementary material for: Why, when and how to update a meta-ethnography qualitative synthesis
Source: Syst Rev. 2016 Mar 15;5:44. doi: 10.1186/s13643-016-0218-4 (PMC4791806; doi:10.1186/s13643-016-0218-4)
Supplement: Additional file 1: Table S1. — Fit between concepts in new papers and synthesised concepts from original meta-ethnography. [file 13643_2016_218_MOESM1_ESM.docx]

**Table S1. Fit between concepts in new papers and synthesised concepts from original meta-ethnography**

| **Included article** | **Final synthesised concepts** | | | | | |
| --- | --- | --- | --- | --- | --- | --- |
|  | **Uncertainty and waiting** | **Disruption to daily life** | **The diminished self** | **Making sense of the experience** | **Sharing the burden** | **Finding a path** |
| Dooks, P., McQuestion, M., Goldstein, D., & Molassiotis, A. (2012). Experiences of patients with laryngectomies as they reintegrate into their community. *Supportive Care in Cancer*, *20*(3), 489-498. | Fear of recurrence | Altered airway, communication, eating, coping with illness / trying to live life, retiring or giving up work | Loss of what was, body image, grief, stigma of association with smoking / being responsible for causation | Implicit across all themes.  No choice – surgery or death | Need empathetic understanding (‘making me feel human’), community health care professionals (HCPs) not having knowledge, peer support, internet/skype | Transitions in recovery, Don’t give up, can overcome, knowing how using peer support, |
| Griffiths, M. J., Humphris, G. M., Skirrow, P. M., & Rogers, S. N. (2008). A qualitative evaluation of patient experiences when diagnosed with oral cancer recurrence. *Cancer nursing*, *31*(4), E11-E17. | Uncertainty, fear etc | Reactions to diagnosis, Life changes and restrictions | Loss of function, emotional vulnerability, withdrawal of friends | Re-evaluation, re-appraisal, thinking about others rather than self | Support/assistance, improvement in relationships | Active coping strategies e.g. hope, taking things day by day, enhanced future – improvement in relationships, examples of enhanced self |
| Semple, C. J., Dunwoody, L., George Kernohan, W., McCaughan, E., & Sullivan, K. (2008). Changes and challenges to patients’ lifestyle patterns following treatment for head and neck cancer. *Journal of advanced nursing*,*63*(1), 85-93. | Concerns about cancer – uncertainty and fears of recurrence | Physical changes – appearance, speech, shoulder dysfunction etc. and how these affect ability to work, finances | Physical changes including appearance ‘I look older now…’ | Re-evaluation – work and day-to- day tasks, looking for physical improvements | (Improvement in) interpersonal relationships | Coping with cancer following treatment, social functioning, enhanced interpersonal relationships, wanting more information about benefits |
| Björklund, M., Sarvimäki, A., & Berg, A. (2008). Health promotion and empowerment from the perspective of individuals living with head and neck cancer. *European journal of oncology nursing*, *12*(1), 26-34. |  |  | As with Ruf et al, examples of enhanced rather than diminished self. | Dialogue with inner self, reappraise life | Social network – emotional and practical support | Ability to regain control and empower oneself  Being enabled through contact with environment (nature); practicing hobbies & activities & contact with a social network. Recognising and embracing existentiality – being totally present in here & now. Reappraise life.  Managing disruption through self-management.  Transformed & improved self-esteem through prioritizing strength & establishing boundaries (Note: this involved putting a limit on one’s social world); not acting as victim. |
| Björklund, M., Sarvimäki, A., & Berg, A. (2010). Living with head and neck cancer: a profile of captivity. *Journal of Nursing and Healthcare of Chronic Illness*, *2*(1), 22-31. | Feelings of existential disequilibrium, living on a rollercoaster | Confined in rogue body (necessity of restrictive living), fear of choking during sleep, caught in permanent illness trajectory, employer threatening legal proceedings, financial problems due to low sick pay;  changed relationships with spouse, isolation. | Hostage, locked up, confined in rogue body, trapped in alien body, health care governing life, being a guinea pig, at mercy of HCPs, depreciated sense of how others see you,  rejection by next of kin, disregarded by HCPs in treatment decisions, lack of accessibility and continuity in health care. | Altered sense of affiliation – reevaluation. | Forced dependency on others, confidence in health care, need to meet ‘soul mates’ (peers with HNC). | Taking actions to explore new life conditions, death as transition, value life and self-image positively (reconciliation with illness), good support & relationships from next of kin are motivation for living, prioritizing self-care over socialising without guilt, preparing for own death, death as transition (no fear of dying) |
| Röing, M., Hirsch, J. M., Holmström, I., & Schuster, M. (2009). Making new meanings of being in the world after treatment for oral cancer. *Qualitative health research*, *19*(8), 1076-1086. | Oneself as transformed | Self as captive to a changed mouth, facial disfigurement and pity - Problems with eating and drinking, learning to live with changes | Changed self | New meanings of being in the world, re-evaluating – importance of family for example | Focusing on loving friends and family | Moving on from oneself as transformed. Oneself as dependent and depended upon – if you have children you have to keep going. |
| Semple, C. J., & McCance, T. (2010). Experience of parents with head and neck cancer who are caring for young children. *Journal of advanced nursing*,*66*(6), 1280-1290. | Living with uncertainty |  | Not being able to fulfill usual roles | Helping children to make sense of the experience; fathers spending more time with children because of having cancer. | Support networks.  Fear of telling the children, other members of family taking on roles, sharing diagnosis with school teachers | Increased appreciation of life. Managing disruption and impact on family |
| Tong, M. C. F., Lee, K. Y. S., Yuen, M. T. Y., & Lo, P. S. Y. (2011). Perceptions and experiences of post‐irradiation swallowing difficulties in nasopharyngeal cancer survivors. *European journal of cancer care*, *20*(2), 170-178. | Fear of recurrence | Dry mouth, difficulty eating, dental problems, choking | Reluctance to explain things to friends, reduced socialisation, impact on work and family |  |  | Self-management strategies for eating difficulties |
| Hu, T. W., Cooke, M., & McCarthy, A. (2009). A qualitative study of the experience of oral cancer among Taiwanese men. *International journal of nursing practice*, *15*(4), 326-333. |  | Challenges to everyday activities e.g. eating, work Immediate reactions to diagnosis. | Loss of life aspiration, feelings of diminished self | Explaining oral cancer through smoking, drinking, betel. | Changes in relationships | Reprioritisation, adapting |
| Thambyrajah, C., Herold, J., Altman, K., & Llewellyn, C. (2010). “Cancer doesn't mean curtains”: Benefit finding in patients with head and neck cancer in remission. *Journal of psychosocial oncology*, *28*(6), 666-682. |  | Eating problems |  | Core focus of paper | New and improved relationships | New appreciation for life, hobbies etc, prioritising friends and family, acceptance of changed self, comfort in religion  Self-management strategies – becoming more health conscious. |
| McQuestion, M., Fitch, M., & Howell, D. (2011). The changed meaning of food: Physical, social and emotional loss for patients having received radiation treatment for head and neck cancer. *European journal of oncology nursing*,*15*(2), 145-151. | Uncertainty about how long symptoms and problems will persist | Eating problems | Loss of ability to eat, changed meaning of food, loss of normality, body image, social loss  Not admitting to eating difficulties to family. |  |  | Changed perspective about food |
| Konradsen, H., Kirkevold, M., & Zoffmann, V. (2009). Surgical facial cancer treatment: the silencing of disfigurement in nurse–patient interactions. *Journal of advanced nursing*, *65*(11), 2409-2418. |  | Disfigurement | Body image  Collusion – nurses assuming disfigurement isn’t that big an issue, not addressing it because they think it isn’t the right time – minimising the problem was a barrier to communication | ‘Disfigurement is a luxury’ |  |  |
| Björklund, M., Sarvimäki, A., & Berg, A. (2009). Health promoting contacts as encountered by individuals with head and neck cancer. *Journal of Nursing and Healthcare of Chronic Illness*, *1*(3), 261-268. |  |  | Symptoms not believed by HCPs/ problems ignored. Dominating or inconsiderate HCPs. Sometimes patients do share but are not acknowledged, self-diagnosis not believed. Problems resulting from treatments ignored |  | Being believed in one’s illness story important through whole trajectory of care..  Having a good working relationship with HCPs. Despair over dominating or inconsiderate HCPs.  The need & desire for receiving individualized tailored care – frustration with health care system bureaucracy. | Knowing how to manage disruption to daily life. Importance of and quality of contact with HPs for managing problems. |
| Foxwell, K. R., & Scott, S. E. (2011). Coping together and apart: exploring how patients and their caregivers manage terminal head and neck cancer. *Journal of psychosocial oncology*, *29*(3), 308-326. |  |  |  | Acceptance of cancer & its consequences including death through belief in fate or God | Coping through support from HCPs-asking for and receiving their help.  Sharing with social network and coping together as a couple; coping through support from social network – getting instrumental, informational and emotional support | Place the problem within a bigger context.  Preparation for death.  Using internal resources to cope  Limiting one’s focus to the present.  Fighting cancer.  Managing & coping- underestimating the seriousness of the problem.  Prioritizing the needs of one’s partner.  Self-management- Avoiding disruption via ‘acceptance’ or managing disruption by avoiding thinking about HNC, maintaining sense of control, investing in areas of life not subsumed by cancer. |

**Key**:

Blue font - adds new dimension to final synthesised concept from original meta-ethnography

Red font – appears to contradict final synthesised concept from original meta-ethnography

Black font - supports final synthesised concept from original meta-ethnography
